# Cerebral Activity to Opposite-Sex Voices Reflected by Event-Related Potentials

**DOI:** 10.1371/journal.pone.0094976

**Published:** 2014-04-11

**Authors:** Ya Li, Feng Gu, Xiliang Zhang, Lizhuang Yang, Lijun Chen, Zhengde Wei, Rujing Zha, Ying Wang, Xiaoming Li, Yifeng Zhou, Xiaochu Zhang

**Affiliations:** 1 CAS Key Laboratory of Brain Function and Disease, School of Life Sciences, University of Science and Technology of China, Hefei, Anhui, China; 2 School of Humanities & Social Science, University of Science & Technology of China, Hefei, Anhui, China; 3 Department of Medical Psychology, Anhui Medical University, Hefei, Anhui Province, China; University of Florida, United States of America

## Abstract

Human voice is a gender discriminating cue and is important to mate selection. This study employed electrophysiological recordings to examine whether there is specific cerebral activity when presented with opposite-sex voices as compared to same-sex voices. Male voices and female voices were pseudo-randomly presented to male and female participants. In Experiment 1, participants were instructed to determine the gender of each voice. A late positivity (LP) response around 750 ms after voice onset was elicited by opposite-sex voices, as reflected by a positive deflection of the ERP to opposite-sex voices than that to same-sex voices. This LP response was prominent around parieto-occipital recording sites, and it suggests an opposite-sex specific process, which may reflect emotion- and/or reward-related cerebral activity. In Experiment 2, participants were instructed to press a key when hearing a non-voice pure tone and not give any response when they heard voice stimuli. In this task, no difference were found between the ERP to same-sex voices and that to opposite-sex voices, suggesting that the cerebral activity to opposite-sex voices may disappear without gender-related attention. These results provide significant implications on cognitive mechanisms with regard to opposite-sex specific voice processing.

## Introduction

Gender discriminating characteristics, such as face, body, voice, and odor, are important cues for mate selection [1]. The perception and recognition of opposite-sex cues may recruit specific neural assemblies different from those recruited when same-sex cues are perceived and recognized. Of particular interest is the reward system, whose activation may drive heterosexuals to be attracted to people of the opposite-sex. This assumption is verified by functional neuroimaging, behavioral and electrophysiological studies. For example, in heterosexuals, cerebral cortices that are activated by opposite-sex faces are different from those activated by same-sex faces. This is especially observed in the reward circuitry of the human brain; findings consistent with results in behavioral studies, which indicate that opposite-sex faces have reward value [2], [3]. Further, an electrophysiological study by Proverbio et al. [4] found that a negative brain response of around 400 ms and a late positive brain response of around 700 ms were elicited when heterosexuals viewed opposite-sex faces as compared to same-sex faces, which may reflect that there is reward-related brain activity when presented with opposite-sex faces.

Human voice is another gender discriminating cue that has attracted attention of researchers [5], [6]. Similar with opposite-faces, opposite-sex voice perception in heterosexuals may also recruit specific neural assemblies, different from those recruited by perception of same-sex voice. Sokhi et al. [7] found that in male participants the perception of female voices resulted in greater activation of the right anterior superior temporal gyrus (STG) than perception of male voices. However, because only male participants were recruited in Sokhi et al.'s study, this right STG activation may have reflected a gender-perception-related activation rather than an opposite-sex specific activation. In order to test this hypothesis, Lattner et al. conducted a study which used a mixed sample of 8 male and 8 female participants. Lattner et al.[8] also found a right STG activation to female voices compared with male voices, but no opposite-sex effect was found. On the contrary, Junger et al. [9] recently found that opposite-sex voices activated specific brain areas including the orbitofrontal cortex (OFC), the middle temporal gyrus (MTG), and the medial prefrontal cortex (MPFC). It is worth noting that the experimental tasks used in these two studies were different. In Junger et al. 's study, the participants were instructed to determine the gender of each voice stimulus, whereas in Lattner et al.'s study, the participants were instructed to determine the naturalness of each voice stimulus. We think that this difference might be the reason for the conflicting results, i.e., opposite-sex voice specific processing may not be an automatic processing but a controlled processing that relies on the focal attention to the gender of the voices.

In the present study, we used electrophysiological recordings to examine whether there are specific neural activities to opposite-sex voices relative to same-sex voices. The event-related potential (ERP) elicited by opposite-sex voices was compared with that elicited by same-sex voices. We hypothesized that due to the importance of opposite-sex voices in mate selection and evolution, it was possible that the opposite-sex voice processing was shaped by evolution, and that this would result in different patterns between ERP to same-sex voices and that to opposite-sex voices. The difference may occur in early latency because previous ERP studies have observed an early voice-specific response (VSR) at around 320 ms after the voice onset which reflects voice specific processing as compared to other sounds [10], [11]. The difference may also occur in late latency; a reflection of high cognitive processing due to activation of emotion and/or reward systems by the reward value of the opposite-sex voices [12]. We also hypothesized that attention may influence the processing of opposite-sex voices as revealed by previous fMRI studies [8], [9]. To measure this influence, we performed two tasks; an explicit task which instructed participants to determine the gender of each voice stimulus (Experiment 1), and an implicit task which instructed participants to press a key when hearing a non-voice stimulus and to ignore the voice stimuli (Experiment 2).

## Materials and Methods

### Ethics Statements

The experiments were conducted in line with principles expressed in the Declaration of Helsinki and the research was approved by the Biomedical Research Ethics Committee of the University of Science and Technology of China. All participants provided informed written consent.

### Experiment 1

#### Participants

Nineteen male and nineteen female participants took part in Experiment 1. All participants were right-handed [13], and self-reported heterosexuals. Male and female participants were matched in age (Male: range: 21–28, mean = 23.62, SD = 1.51; Female: range: 18–27, mean = 23.23, SD = 2.06) and years of education (Male: mean = 17.00, SD = 1.63; Female: mean = 16.74, SD = 1.59).

#### Stimuli

**Figure 1:**
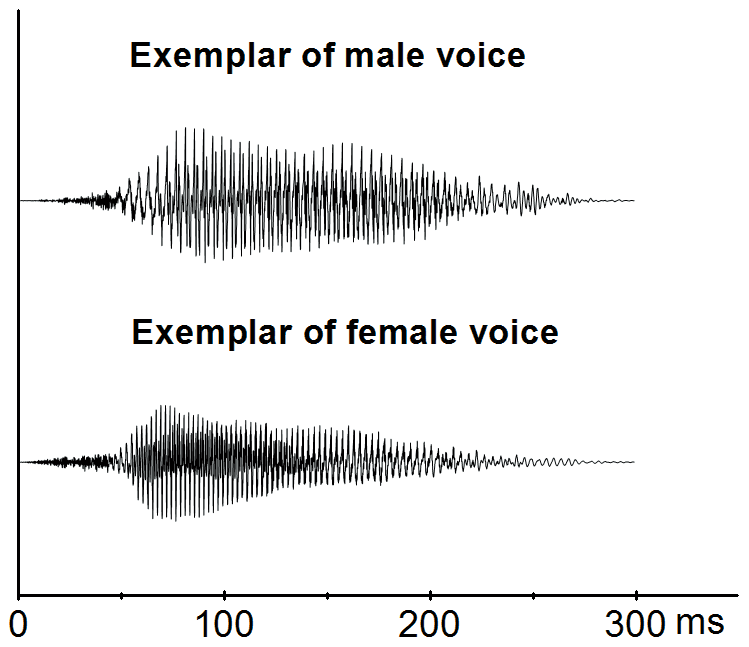
Sound waveforms. Two representative exemplars of the 9 male voices and 9 female voices were illustrated. The consonant part (/h-/) of the sound was normalized at around 40 ms in duration and the whole sound was normalized at 300 ms in duration.

The original voice sounds were digitally recorded from nine young male speakers and nine young female speakers (age ranged from 18–26 years) in a soundproof studio with a 48-kHz sampling frequency and 16-bit quantization. Chinese monosyllabic word/hei4/(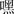 in Chinese, whose pronunciation and meaning are similar with the English word “hey”) was chosen. The speakers were asked to articulate this syllable several times with neutral emotion, and the well pronounced ones were chosen for further processing. The consonant part (/h-/) of the sound was normalized around 40 ms in duration and the whole sound was normalized to 300 ms in duration. The sound waveforms of a representative exemplar of male voices and an exemplar of female voices are illustrated in Figure 1.

A gating test was performed to determine the gender recognition point of each voice stimulus. The gender recognition point refers to the point in time at which the gender of the voice can actually be recognized. For each voice stimulus, fragments of the initial 30, 40, 50, 60, 70, 80, 90, and 100 ms of each stimulus were presented to the participants in a gradually increasing order. Participants were asked to indicate whether they could identify the gender of the voice. The test began with 30-ms fragments, and the voice length was increased until the participant could ascertain the gender of the voice by giving an oral report. Ten participants (5 males and 5 females) took part in this test (these participants did not participate in the EEG experiments discussed below). The results indicated that the gender of each voice could be correctly recognized in at least 9 participants by 50-ms or 60-ms fragments, suggesting that the gender recognition point of each voice was around 50–60 ms after the voice onset.

#### Procedure

**Figure 2:**
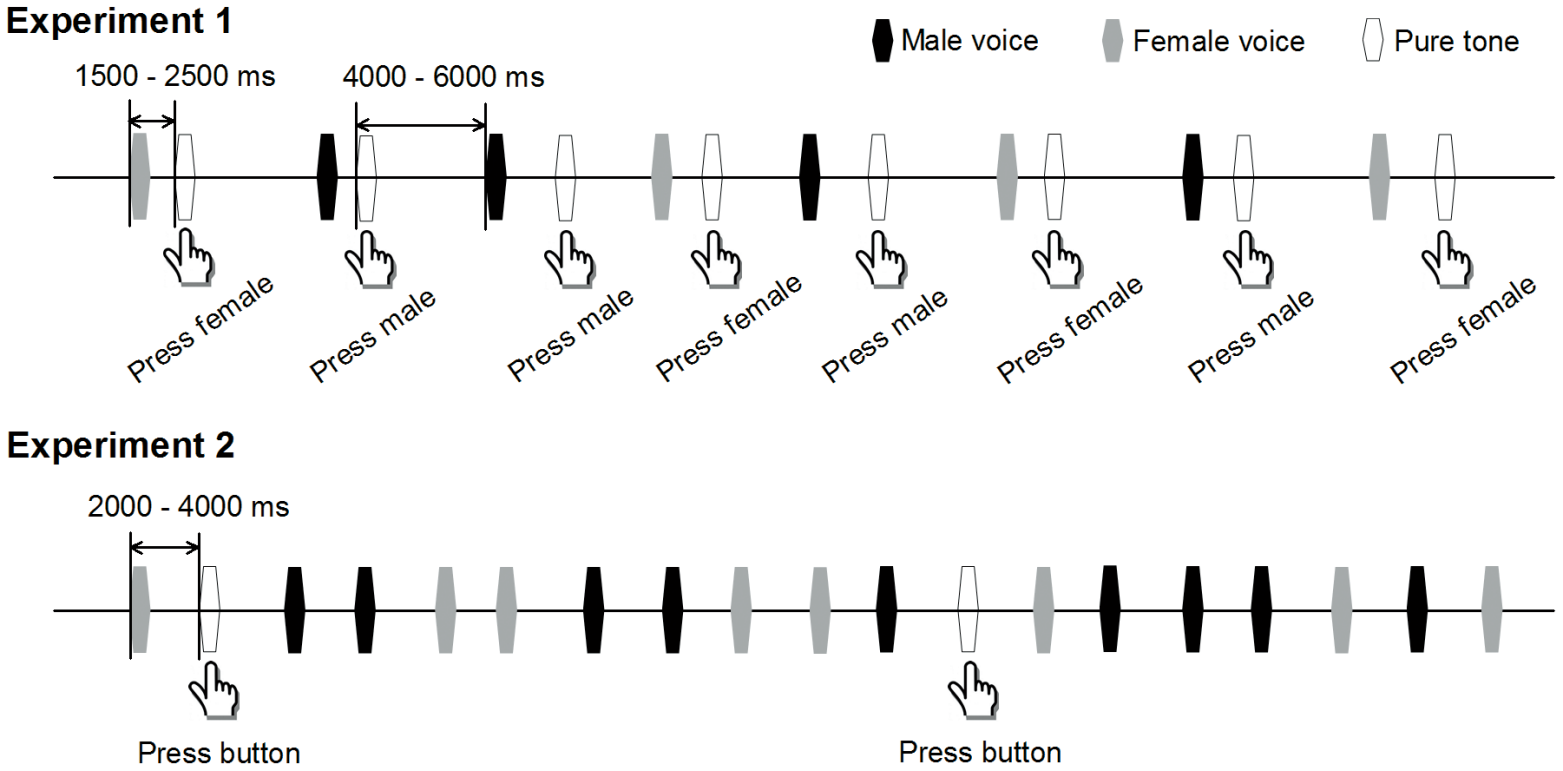
Experimental paradigms. In Experiment 1, the 9 male voices and 9 female voices were pseudo-randomly presented to participants with equal probability. A pure tone sound was presented after each voice. Participants were instructed to press the female or male key when hearing the pure tone to indicate the gender information of the voice just before the pure tone. In Experiment 2, the 9 male voices and 9 female voices, and a pure tone stimulus were pseudo-randomly presented to participants. Participants were instructed to press the key when hearing the pure tone stimulus.

The 9 male and 9 female voices were pseudo-randomly presented to participants with equal probability through headphones (Sennheiser HD 25 II), and two adjacent identical voices were separated by at least two other voices. A pure tone sound (600 Hz, 300 ms, 70 dB SPL) was presented 2000 ms (mean, range from 1500 to 2500 ms) after the onset of each voice. The next voice was presented 5000 ms (mean, range from 4000 to 6000 ms) after the onset of the pure tone. Participants were seated on a reclining chair and were instructed to press the female or male key using their right index or middle finger (the key mapping was counterbalanced between participants) after hearing the pure tone to indicate whether the gender of the voice played just before the pure tone was female or male (Figure 2). Participants were instructed to press the button after the pure tone sound rather than immediately after the voice sound to avoid possible influence of press-related potential which may contaminant voice-related potential. Four blocks were presented to each participant and there was a 5 minute break between blocks. Each block consisted of 108 voices (54 male and 54 female voices), and lasted for about 756 s. The experiment ran for approximately 2 hours per participant. This time included the time for electrode application and removal.

#### EEG Recording

EEGs were recorded using SynAmps 2 amplifier (NeuroScan, Charlotte, NC, U.S.) with a cap carrying 64 Ag/AgCl electrodes placed on the scalp at specific locations according to the extended international 10–20 system. The electrical activities were recorded over left and right mastoid. Horizontal electrooculography (EOG) was recorded using bipolar channel placed lateral to the outer canthi of both eyes, and vertical EOG was recorded using bipolar channel placed above and below the left eye. The reference electrode was attached to the tip of the nose and the ground electrode was attached to AFz. Impedance between the reference electrode and any recording electrode was kept under 5 kΩ. Alternating current signals (0.03–100 Hz) were continuously recorded and digitized with a 24 bit resolution at a sampling rate of 500 Hz.

#### Data analysis

The vertical EOG artifacts were corrected using a regression-based procedure [14]. Then the EEG data in the whole-head recordings were off-line band-pass (1–25 Hz) filtered with a finite impulse response (FIR) filter. Epochs were set to 1000 ms in length, including a 100 ms pre-stimulus baseline. Epochs with amplitudes exceeding ±50 µV at any channel except for EOG channels were excluded from averaging. ERPs to the 9 male and 9 female voices were averaged from the remaining epochs and corrected relative to the baseline (100 ms pre-stimulus mean amplitude). For each participant, the ERP to same-sex voices and the ERP to opposite-sex voices were obtained by grand-averaging the ERP to the 9 female voices or 9 male voices. Then a cluster-based permutation test was performed using BESA Statistics software (ver. 1.0) to test whether there was significant difference between the ERP to same-sex voices and the ERP to opposite-sex voices. Firstly, a preliminary paired sample *t* test was performed between ERP to same-sex voices and that to opposite-sex voices at each sampling point through 0–900 ms at each electrode site. Electrode sites are considered to be adjacent if their distance is less than 4 cm. Clusters were identified as where spatially and temporally adjacent sampling points reached significant value (2-tailed, p<0.05) in the paired sample *t* test. Secondly, a parameter-free cluster permutation statistics was performed using a cluster alpha level of *p*<0.05. The sum of the *t* values within a cluster was used as cluster-level statistic. A reference distribution from 1000 random sets of permutations was created. The cluster *p* value was estimated as the proportion of the elements in the randomization distribution exceeding the cluster-level statistic [15].

### Experiment 2

#### Participants

Nineteen male and nineteen female participants took part in Experiment 2. None of them participated in Experiment 1. All participants were right-handed [13], self-reported heterosexuals. Male and female participants were matched in age (Male: range: 20–31, mean = 24.05, SD = 2.53; Female: range: 19–26, mean = 22.94, SD = 2.15) and years of education (Male: mean = 17.11, SD = 2.00; Female: mean = 16.47, SD = 1.95).

#### Stimuli and procedure

The stimuli used in Experiment 2 were the same as those used in Experiment 1. The 9 male voices and 9 female voices, and a pure tone stimulus (600 Hz, 300 ms, 70 dB SPL) were pseudo-randomly presented to participants with a stimulus onset asynchrony of 3000 ms (range from 2000 to 4000 ms). These 19 sounds were presented with equal probability (1/19), and two adjacent identical sounds were separated by at least two sounds. Participants were seated on a reclining chair and were instructed to press a key after hearing the pure tone stimulus (Figure 2). Two blocks were presented to each participant and there was a 5 minute break between blocks. Each block consisted of 247 sounds, and lasted for about 740 s. The experiment ran for approximately 1.5 hours per participant. This time included the time for electrode application and removal.

#### EEG Recording and analysis

The settings for the EEG recordings were the same as in Experiment 1, and EEG data were analyzed following the procedures described in Experiment 1.

## Results

### Experiment 1

The behavioral results indicated that 99.61% (SD = 0.13%) of the male voices and 99.90% (SD<0.01%) of the female voices were correctly recognized by female participants, and 99.12% (SD = 0.01%) of the male voices and 98.64% (SD = 0.02%) of the female voices were correctly recognized by male participants. For female participants, the press latency to the pure tone after male voices was 658.9 ms (SD = 23.8 ms), and to the pure tone after female voices was 660.2 ms (SD = 24.3 ms). For male participants, the press latency to the pure tone after male voices was 732.1 ms (SD = 22.5 ms), and to the pure tone after female voices was 705.0 ms (SD = 22.4 ms). Two-way ANOVA with group gender (female and male participants) as between-group factor and voice gender (male and female voices) as within-group factor was performed to analyze the peak latency difference. No main effect and no significant interaction were found (*p* values>0.19).

**Figure 3:**
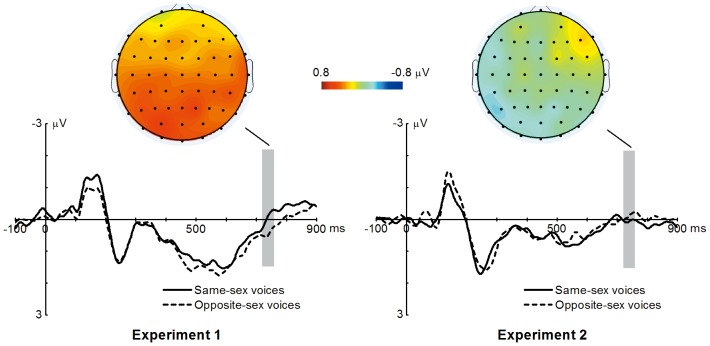
Grand-averaged ERP to opposite-sex voices and that to same-sex voices at electrode site PO3. In Experiment 1, the ERP to opposite-sex voices is positively deflected than that to same-sex voices at parieto-occipital recording sites (cluster *p* value = 0.014). In Experiment 2, there is no significant difference between the ERP to same-sex voices and that to opposite-sex voices. Gray bars represent the time-window (720–760 ms) centered on the peak of the difference between the ERP to same-sex voices and that to opposite-sex voices in Experiment 1. The topographies illustrate the difference between the ERP to same-sex voices and that to opposite-sex voices at the time-window (720–760 ms).

The cluster permutation test revealed that the ERP to opposite-sex voices was positively deflected than that to same-sex voices at parieto-occipital recording sites (cluster *p* value = 0.014), and was around 750 ms after the voice onset (i.e., around 700 ms after the gender recognition point). Electrode PO3 was selected for illustration because the maximal difference between the ERP to same-sex voices and that to opposite-sex voices was at 740 ms at the electrode site PO3 (Figure 3). In order to examine whether the opposite-sex effect was significant in both male and female participants, the cluster permutation test was performed in male and female participants separately. The results of neither the male group nor the female group showed any significant difference between the ERP to same-sex voices and that to opposite-sex voice. The absence of significance might be caused by the fact that the opposite-sex effect is weak, and that the samples were reduced to half when the male and female participants were separated. Therefore, we used a statistically less rigorous method to examine this question by choosing an electrode and a time window that showed the maximal difference between the ERP to same-sex voices and that to opposite-sex voices as the region of interest. Then the mean ERP amplitude within this region was separately analyzed in male and female participants. Because the maximal difference between the ERP to same-sex voices and that to opposite-sex voices was at 740 ms at the electrode site PO3, for each participant, the mean ERP amplitude within the 40 ms time window centered at 740 ms at PO3 was separately calculated for the ERP to same-sex voices and that to opposite-sex voices. Planned pair-wise *t* test was performed between the mean ERP amplitude to same-sex voices and that to opposite-sex voices separately for the male and female groups. For both groups, the ERP to opposite-sex voices was marginally larger than that to same-sex voices (*t* (18) = 1.872, *p* = 0.078, 2-tailed; *t* (18) = 1.780, *p* = 0.092, 2-tailed). Therefore, the opposite-sex effect in current study was not from one specific gender group.

### Experiment 2

The behavioral results showed that 100% of the pure tones were correctly pressed by all male and female participants, indicating that the task was very easy for them. The press latency of female participants was 596.3 ms (SD = 14.5 ms), and that of male participants was 654.0 ms (SD = 23.5 ms). Independent sample *t* test was performed and the results indicated that there was no significant press latency difference between male and female participants (t = 0.908, p = 0.370).

The cluster permutation test did not find any significant difference between the ERP to same-sex voices and that to opposite-sex voices.

In Experiment 1, participants' particular attention was placed on gender of the voices whereas the gender of the voices was ignored in Experiment 2. In order to examine whether such attention to gender information of the voices significantly affected the opposite-sex voice processing, the ERP to the same-sex voices was subtracted from that to the opposite-sex voices for each participant and each experiment. The resulting ERP which reflected the opposite-sex specific response was analyzed by cluster-based permutation test to examine whether there was significant difference between Experiment 1 and Experiment 2. The result did not reveal any significant differences.

## Discussion

In this study, we found that a late positivity (LP) response around 750 ms after voice onset was elicited by opposite-sex voices, as reflected by a positive deflection of the ERP to opposite-sex voices than that to same-sex voices. Since the gender recognition point of our voice stimuli was 50–60 ms, the LP to opposite-sex voices peaked at around 700 ms after the gender recognition point. Further, this LP response to opposite-sex voices was statistically significant in the explicit gender discriminating task (Experiment 1) and was not prominent in the implicit task (Experiment 2).

The LP response to opposite-sex voices is consistent with results of studies on opposite-sex faces, which have shown a similar latency (∼700 ms) and topography (prominent at parieto-occipital electrode sites) of LP response to opposite-sex faces [4]. Moreover, the LP was elicited by attractive or emotional faces [16], [17], and emotional pictures [18]. Similar LP responses were also elicited by smoking cues in smokers [19], [20], and drug cues in heroin dependent participants [21]. These addictive-drug related cues are able to elicit reward and emotion processes [22]. Therefore, the LP response in the present study very likely represented emotion and/or reward cerebral activities to opposite-sex cues. This is in line with results from behavioral studies that heterosexuals are attracted to and prefer opposite-sex faces and voices to those of the same-sex [12], [23], [24]. FMRI studies have also found that emotion and reward cerebral cortices are activated by opposite-sex cues in response to both opposite-sex voices and faces [3], [9]. This is especially the case with OFC, which is known to be involved in the evaluation of emotional information in both voice and face stimuli [25]. Further studies should be conducted to examine whether the LP response is a reflection of OFC activity.

Male and female voices usually differ in fundamental frequency, formant frequencies, and other sexually dimorphic acoustical cues [26], [27]. Male and female voices elicit distinct neural activities due to their acoustic differences [7], [8], [28]. Even when these acoustic differences are controlled, cerebral activity differences in voice processing still persist [8], [29]. The gender-recognition-related cerebral activities do not differ between male and female participants, thus, they do not represent any opposite-sex specific processing [8], [29]. Since the male and female participants as well as the male and female voices were well balanced in this study, acoustics and gender are matched between opposite-sex and same-sex voices. Therefore, the LP found in the study could be reasonably explained as a bias for opposite-sex voice processing rather than acoustic or gender processing of voices.

Human voices are thought to be specifically processed at STS [30]. Some ERP studies have found that this specific voice processing takes place at around 320 ms after the voice onset, named the VSR and is negatively deflected as compared to non-voice sounds [10], [11]. In this study, we did not find any opposite-sex specific brain potential around the VSR latency. We believe that although the acoustic relevant information of gender perception is processed at the anterior part of the STS [29], the opposite-sex specific processing may be a high level cognitive process that involves emotion and reward mechanisms. This is in line with results of fMRI studies that found the activation of fronto-temporal neural network in response to opposite-sex voices but did not find any activation around STS [9].

As mentioned at the outset of the paper, Junger et al.'s study, which used an explicit gender discriminating task, found opposite-sex specific cerebral activity including emotion and/or reward cortices [9]. On the contrary, Lattner et al.'s study, which used an implicit task, did not find any opposite-sex effect [8]. Ethofer et al. [31] used a passive listening task and did not find any enhanced activation of emotion and reward cortices to opposite-sex voices even using erotic voices. Therefore, attention might have an influence on specific opposite-sex processes. This is partially supported by this study, as the results in this study have indicated that the LP is significant in the explicit gender discriminating task (Experiment 1) but not present in the implicit task (Experiment 2). However, direct comparing between Experiment 1 and Experiment 2 did not reveal significant difference. Therefore, this hypothesis that attention affects opposite-sex voice processing is not strongly supported by statistics in current study. Further evidence would be needed in the future study.

However, in visual modality, opposite-sex faces elicit specific cerebral activity even in implicit task experiments [3], [4]. Gender categorization studies have suggested that the processing of faces dominate over the processing of voices [32], and that there is bias towards faces as compared to voices in conveying emotional information [32]–[34]. Further, these studies have also revealed that opposite-sex faces not only do elicit LP response but also elicit an earlier negative response around 400 ms (N400) after face onset. This N400 response was not found in our voice study. The N400 response demonstrates a significant earlier opposite-sex discrimination time in visual process than that in auditory process, suggesting the opposite-sex cue conveyed by face may be more effective or faster than that conveyed by voice in activating emotion and reward system. This further suggests a potential different mechanism related to gender information processing between visual and auditory pathways. Certainly, this speculation presents an interesting hypothesis for further studies in future.

Our results lead to a range of implications for the cognitive mechanisms of opposite-sex specific voice processing. Firstly, the absence of opposite-sex effect in the early/VSR latency, and the absence of opposite-sex effect in the implicit task, suggest that the opposite-sex specific voice processing is likely not an automatic mechanism. Although human voices are specifically and automatically processed at STS and reflected by the VSR [10], [11], [30], the opposite-sex voices are not particularly processed at this stage. This is irrespective of the importance of the opposite-sex cues for mate selection.

Secondly, the opposite-sex effect is relatively late (∼700 ms), suggesting that the opposite-sex effect is a controlled process at high cognitive level. We infer that this process is an emotion and/or reward related process due to the reward value of the opposite-sex voices that is reported by behavioral study [12].

Thirdly, the opposite-sex effect was not found in our implicit task, whereas the opposite-sex effect was prominently observed in the implicit task in face studies [4]. It would be reasonable to conclude that voice cues may not be as effective as face cues in eliciting reward activities, and this may further imply that voice cues may not be as important as face cues in mate selection during human evolution.

Lastly, the LP response which reflected the opposite-sex effect in this study is similar with many previous studies that found the LP-like response elicited by reward cues [4], [16]–[21]. This suggests that the LP response may reflect a common neural basis across different reward cues including gender, aesthetics, smoking, drug-use, etc.
